# Impact of chemotherapy, radiotherapy, and endocrine therapy on sick leave in women with early-stage breast cancer during a 5-year period: a population-based cohort study

**DOI:** 10.1007/s10549-020-05720-4

**Published:** 2020-06-06

**Authors:** Anna Plym, Anna L. V. Johansson, Hannah Bower, Anna-Karin Wennstig, Irma Fredriksson, Johan Ahlgren, Mats Lambe

**Affiliations:** 1 Department of Medical Epidemiology and Biostatistics, Karolinska Institutet, PO Box 281, 171 77 Stockholm, Sweden; 2 Cancer Registry of Norway, Oslo, Norway; 3 Department of Surgical and Perioperative Science, Umeå University, Umeå, Sweden; 4 Department of Oncology, Sundsvall Hospital, Sundsvall, Sweden; 5 Department of Molecular Medicine and Surgery, Karolinska Institutet, Stockholm, Sweden; 6 Department of Breast and Endocrine Surgery, Karolinska University Hospital, Stockholm, Sweden; 7 Regional Cancer Center, Uppsala University Hospital, Uppsala, Sweden; 8 Department of Oncology, Faculty of Medicine and Health, Örebro University, Örebro, Sweden

**Keywords:** Breast cancer, Chemotherapy, Disability pension, Endocrine therapy, Radiotherapy, Sick leave

## Abstract

**Purpose:**

To examine the influence of type of oncological treatment on sick leave in women of working age with early-stage breast cancer.

**Methods:**

We identified 8870 women aged 30–64 diagnosed with stage I–II breast cancer between 2005 and 2012 in the Breast Cancer Data Base Sweden. Associations between type of oncological treatment (radiotherapy, endocrine therapy, and chemotherapy) and sick leave were estimated by hazard ratios, probabilities, and length of sick leave using multi-state survival analysis.

**Results:**

During the first 5 years after diagnosis, women aged 50–54 years at diagnosis receiving chemotherapy spent on average 182 (95% CI 151–218) additional days on sick leave compared with women not receiving chemotherapy, but with otherwise similar characteristics. Correspondingly, women initiating endocrine therapy spent 30 (95% CI 18–44) additional days on sick leave and women receiving post-mastectomy radiotherapy 53 (95% CI 37–69) additional days. At year five, the rate of sick leave was increased in women who had received chemotherapy (HR 1.19, 95% CI 1.11–1.28) or endocrine therapy (HR 1.15, 95% CI 1.05–1.26). Chemotherapy and endocrine therapy were associated with increased rates of sick leave due to depression or anxiety.

**Conclusion:**

Our findings of increased long-term risks of sick leave after oncological treatment for breast cancer warrant attention from caregivers taking part in cancer rehabilitation. In light of the ongoing debate about overtreatment of early-stage breast cancer, our findings point to the importance of properly selecting patients for chemotherapy not only for the medical toxicity but also the possible impact on their livelihood.

**Electronic supplementary material:**

The online version of this article (10.1007/s10549-020-05720-4) contains supplementary material, which is available to authorized users.

## Introduction

Many women diagnosed with early-stage breast cancer undergo intensive treatment protocols, with combinations of surgery and oncological treatment such as radiotherapy, endocrine therapy, and/or chemotherapy. These treatments are associated with side effects that can influence well-being and restrict daily activities. For women of working age, representing half of all women diagnosed with breast cancer in North America and Europe [1], return to work and ability to work may be negatively affected. Previous studies have demonstrated that diagnosis and treatment for breast cancer are associated with increased rates of sick leave, receipt of disability pension, and other work–life-related events, also beyond the first year after diagnosis [2–7].

Chemotherapy has been identified as a major factor delaying return to work [8], but previous research on the long-term association between chemotherapy and absence from work has been less conclusive. Also after controlling for factors related to stage and tumor biology, chemotherapy has been reported to increase the risk of sick leave, disability pension receipt, and unemployment in some [2, 4, 6], but not all previous studies [5, 7, 9, 10]. Results regarding the association between adjuvant endocrine treatment and absence from work are also conflicting [2, 5, 6, 9, 10], as are some of the studies regarding the influence on radiotherapy on the ability to work [2, 11]. In addition to different study populations and follow-up times, these conflicting results are possibly related to difficulties separating out the treatment-related side effects from the influence of underlying cancer characteristics.

Over-treatment of women with early-stage breast cancer is a concern, possibly leading to unnecessary side effects and prolonged absence from work. Recently, predictive tools based on genetic analyses have been proven successful in identifying women who are likely to benefit from chemotherapy, sparing those unlikely to benefit [12, 13]. In light of those findings and to better understand the consequences of breast cancer treatment on working life, the present study aims to examine and quantify the possible association between receipt of radiotherapy, endocrine therapy, and chemotherapy and sick leave in a large cohort of women with early-stage breast cancer, while controlling for important clinical and sociodemographic factors and accounting for the competing events of disability pension receipt and death.

## Methods

This population-based cohort study included women identified in the Breast Cancer Data Base Sweden (BcBaSE). BcBaSE is an individual-level record-linkage between the Breast Cancer Quality Registers in Stockholm-Gotland, Uppsala-Örebro, and the Northern regions, and several other national health care and demographic registers as previously described [7, 14]. BcBaSE contains detailed information on patient, treatment, tumor characteristics, and cancer outcome for 99% of all women diagnosed with breast cancer within the geographical capture areas, which include around 60% of the Swedish population. For this study, we extracted data from BcBaSE for women diagnosed with invasive breast cancer from July 1, 2005 to December 31, 2012. Further inclusion criteria were that women were aged 30–64 years at diagnosis, had pathological stage I–II breast cancer (according to the UICC TNM classification 7th edition [15]), no prior receipt of disability benefits, and no sick leave one month prior to diagnosis, resulting in a final study population of 8870 women.

The exposure of interest was type of oncological treatment: radiotherapy, endocrine therapy, or chemotherapy. Since nearly all women with breast-conserving surgery had radiotherapy, we were only able to study post-mastectomy radiotherapy. Information on chemotherapy and radiotherapy was extracted from the Breast Cancer Quality Registers, whereas endocrine therapy was defined according to information in the Swedish Prescribed Drug Register, a nationwide register that since July 2005 collects information on filled drug prescriptions from Swedish pharmacies [16]. Adjuvant endocrine therapy was defined as at least one filled prescription of oral tamoxifen or an aromatase inhibitor during follow-up. The median time from diagnosis to initiation of endocrine therapy was 152 [interquartile range (IQR) 72–224] days.

The primary outcome was sick leave as registered in the national database held by the Swedish Social Insurance Agency, which has been linked to BcBaSE. The Swedish Social Insurance Agency grants sickness benefits to all individuals living or working in Sweden in case of reduced work capacity because of disease or injury. In the case of permanently reduced work capacity, the Swedish Social Insurance Agency can grant disability pension. The first 14 days of a sick-leave period are generally reimbursed by the employer and not by the Swedish Social Insurance Agency; thus, sick leave periods < 15 days were not included in the present analysis. Both all-cause and cause-specific sick leave [due to depression and anxiety-related conditions (ICD-10 codes F32–F34, F38–F43, F45, F48), cancer (C00-D48, Z85), or other conditions (all other ICD-10 codes)] were studied. The national database only records the first diagnosis of a sick-leave period, later changes are not captured (around 7% of all diagnoses are later changed to a diagnosis within another diagnostic group) [17]. According to Swedish national breast cancer management guidelines [18], sick leave is often required during adjuvant chemotherapy [typically 126 days (6 × 21 days) of treatment] and can be indicated during radiotherapy [typically 35 days (5 weeks) of treatment].

From the Breast Cancer Quality Registers, we also retrieved information on tumor characteristics and type of surgery. Information on level of education was obtained from the Longitudinal Integration Database for Health Insurance and Labour Market Studies held by Statistics Sweden. Information on the medical history of mental disorders prior to diagnosis was collected from the Patient Register kept by the National Board of Health and Welfare.

### Statistical analysis

Women were followed for the occurrence of sick leave from the date of diagnosis until reaching age 65, disability pension receipt, death, emigration, or end of follow-up (December 31, 2013), whichever came first. To study the impact of treatment type on sick leave while taking all sick-leave periods and the competing events of disability pension receipt or death into account, a multi-state model with four states (work, sick leave, disability pension, and death) and seven transitions was created (Supplemental Fig. 1). Emigration and end of follow-up were treated as censoring events.

We first estimated hazard ratios and 95% confidence intervals (CI) for transitions between sick leave and work (transitions 1 and 2) using proportional hazards flexible parametric survival models with 5 degrees of freedom for the baseline hazard [19, 20]. To examine the time-dependent effect of exposures, we also fitted a non-proportional model for transition 1 including a time-varying effect of all exposures with 3 degrees of freedom.

We further estimated absolute measures of sick leave (probabilities and length of stay) by modeling all seven transitions in the multi-state model with flexible parametric survival models and performed model-based predictions as recently described [20] (See Supplementary Table 1 for model specification). We predicted probabilities of sick leave, disability pension receipt, and death as well as the restricted mean length of stay on sick leave during the first 5 years (1825 days) after diagnosis, together with 95% CI. The latter measures the total number of days spent on sick leave, including weekends, and does not distinguish between part- and full-time sick leave. All predictions were made for specific covariate patterns, holding all other variables than type of oncological treatment constant.

In all models, time since diagnosis was the underlying time-scale and the following variables were included: chemotherapy (yes, no), surgery/radiotherapy (mastectomy, mastectomy + radiotherapy, breast-conserving surgery + radiotherapy), initiation of endocrine therapy (yes, no), tumor size (1–10, 11–20, > 20 mm or 1–30, > 30 mm), lymph node involvement (N0, N1), type of axillary surgery [sentinel node biopsy (SNB) only, axillary lymph node dissection (ALND)], estrogen receptor (ER) status (negative, positive), age at diagnosis (categorized in 5-year intervals), highest level of education [low (≤ 9 years), middle (10–12 years), high (≥ 13 years)], prior sick leave defined as a record of sick leave (> 14 days) in the period 366 to 730 days before diagnosis (yes, no), calendar year of diagnosis (2005–2008, 2009–2012), and region of residence (Stockholm-Gotland, Uppsala-Örebro, the Northern regions). In models used for estimation of absolute measures, we included interaction terms between type of surgery and type of oncological treatment, as well as between chemotherapy and endocrine therapy. All analyses were complete-case analyses.

To study cause-specific sick leave, we extended the multi-state model to include three different states for sick leave (due to depression/anxiety, cancer, or other) (Supplemental Fig. 2). Hazard ratios and 95% CI for transitions between sick leave and work (transitions 1–3) were estimated as described above, with additional adjustment for medical history of mental disorders. Presented hazard ratios should be interpreted as the relative rate of sick leave among women available for work (i.e., women not on sick leave due to any other reason, and not on disability pension receipt or deceased). Absolute measures were predicted using the models specified in Supplementary Table 2.

All analyses were performed using R (version 3.4.1) and STATA software (version 15.0/IC; Stata Corporation, College Station, TX).

The study was approved by the Ethical Review Board in Stockholm, Sweden.

## Results

Of the 8870 women included in the study, 55% had pathological stage I and 45% pathological stage II breast cancer. The median age at diagnosis was 53 years (interquartile range [IQR] 46–60). Breast-conserving surgery was performed in 67% of women, of which nearly all received post-operative radiotherapy (Table 1). Approximately half of all women that underwent mastectomy received radiotherapy. In total, 50% of women received chemotherapy, and 78% initiated treatment with tamoxifen or an aromatase inhibitor.

**Table 1:** Characteristics of women of working age diagnosed with early-stage breast cancer between 2005 and 2012 (*N* = 8870) together with hazard ratios and 95% confidence intervals for sick leave and return to work after sick leave

Characteristic	Women		Sick leave	Return to work after sick leave
	*n*	(%)	HR^a^ (95% CI)	HR^a^ (95% CI)
Chemotherapy				
No	4451	(50)	1 (Ref.)	1 (Ref.)
Yes	4419	(50)	1.28 (1.22–1.34)	0.43 (0.41–0.45)
Radiotherapy (RT)				
Mastectomy only	1337	(15)	1 (Ref.)	1 (Ref.)
Mastectomy + RT	1512	(17)	1.11 (1.04–1.18)	0.81 (0.76–0.87)
BCS + RT	5751	(65)	0.82 (0.78–0.86)	0.91 (0.87–0.96)
BCS only^b^	192	(2)		
Missing	78	(1)		
Endocrine therapy^c^				
No	1982	(22)	1 (Ref.)	1 (Ref.)
Yes	6888	(78)	1.08 (1.00–1.17)	0.84 (0.78–0.91)
Tumor size (mm)				
1–10	2178	(25)	1 (Ref.)	1 (Ref.)
11–20	4154	(47)	1.01 (0.96–1.06)	0.89 (0.85–0.94)
> 20	2453	(28)	1.04 (0.98–1.10)	0.86 (0.81–0.91)
Missing	85	(1)		
Lymph node involvement				
N0	6391	(72)	1 (Ref.)	1 (Ref.)
N1	2479	(28)	1.04 (0.98–1.09)	0.92 (0.87–0.97)
Axillary surgery				
SNB only	4016	(45)	1 (Ref.)	1 (Ref.)
ALND	4362	(49)	1.06 (1.01–1.11)	0.83 (0.79–0.88)
None/missing	492	(6)		
ER status				
ER+	7409	(84)	1 (Ref.)	1 (Ref.)
ER−	1334	(15)	1.11 (1.01–1.21)	0.71 (0.65–0.78)
Missing	127	(1)		
Age at diagnosis				
< 45	1631	(18)	1 (Ref.)	1 (Ref.)
45–49	1583	(18)	0.90 (0.85–0.96)	1.08 (1.02–1.15)
50–54	1655	(19)	0.93 (0.88–0.99)	1.11 (1.05–1.18)
55–59	1779	(20)	0.91 (0.85–0.96)	1.13 (1.06–1.19)
60–64	2222	(25)	0.60 (0.56–0.64)	1.26 (1.18–1.35)
Education				
Low (0–9 year)	962	(11)	1 (Ref.)	1 (Ref.)
Middle (10–12 year)	3760	(42)	1.20 (1.12–1.28)	1.10 (1.02–1.18)
High (> 12 year)	4088	(46)	1.14 (1.06–1.22)	1.09 (1.02–1.17)
Missing	60	(1)		
Prior sick leave				
No	7859	(89)	1 (Ref.)	1 (Ref.)
Yes	1011	(11)	1.56 (1.48–1.65)	0.76 (0.72–0.80)
Region of residency				
Stockholm-Gotland	4233	(48)	1 (Ref.)	1 (Ref.)
Uppsala-Örebro	3402	(38)	1.14 (1.09–1.19)	1.25 (1.20–1.31)
Northern regions	1235	(14)	1.21 (1.15–1.28)	0.90 (0.85–0.96)
Year of diagnosis				
2005–2008	3929	(44)	1 (Ref.)	1 (Ref.)
2009–2012	4941	(56)	0.94 (0.90–0.98)	1.20 (1.15–1.25)

A higher hazard ratio for sick leave means a higher rate of sick leave, whereas a higher hazard ratio for return to work means a higher rate of return to work

*ALND* axillary lymph node dissection, *BCS* breast-conserving surgery, *ER* estrogen receptor, *RT* radiotherapy, *SNB* sentinel node biopsy

^a^Hazard ratios were adjusted for chemotherapy, radiotherapy/surgery, endocrine therapy, tumor size, lymph node involvement, axillary lymph node dissection, ER status, age at diagnosis, level of education, prior sick leave, region of residency, and calendar year of diagnosis. The underlying time-scale was time since diagnosis (the clock-reset approach gave similar estimates)

^b^Not included in the analysis because of small sample size

^c^Initiation of endocrine therapy

All of the studied oncological treatments were associated with an overall increased rate of sick leave and a lower rate of return to work, with the strongest association found for receipt of chemotherapy (Table 1). In analyses examining time-dependent effects, the risk of sick leave after chemotherapy and after post-mastectomy radiotherapy was highest in the first year of diagnosis (Table 2). At year five, the rate of sick leave remained elevated in women who had received chemotherapy (HR 1.19, 95% CI 1.11–1.28), but not in women who had received post-mastectomy radiotherapy (HR 1.06, 95% CI 0.96–1.17). Women initiating endocrine therapy had an increased rate of sick leave from year two onward, with an HR of 1.15 (95% CI 1.05–1.26) at year five. Results were similar when restricting the analysis to women with ER+ tumors only (Supplementary Tables 3 and 4).

**Table 2:** Time-dependent hazard ratios and 95% confidence intervals for sick leave

Type of treatment	Sick leave					
	Month 6	Year 1	Year 2	Year 3	Year 4	Year 5
	HR^a^ (95% CI)	HR^a^ (95% CI)	HR^a^ (95% CI)	HR^a^ (95% CI)	HR^a^ (95% CI)	HR^a^ (95% CI)
Chemotherapy						
No	1 (Ref.)	1 (Ref.)	1 (Ref.)	1 (Ref.)	1 (Ref.)	1 (Ref.)
Yes	1.70 (1.49–1.95)	1.47 (1.34–1.62)	1.25 (1.17–1.33)	1.22 (1.14–1.30)	1.20 (1.13–1.28)	1.19 (1.11–1.28)
Radiotherapy (RT)						
Mastectomy only	1 (Ref.)	1 (Ref.)	1 (Ref.)	1 (Ref.)	1 (Ref.)	1 (Ref.)
Mastectomy + RT	1.23 (0.99–1.53)	1.16 (1.00–1.34)	1.08 (0.98–1.19)	1.07 (0.98–1.17)	1.07 (0.97–1.17)	1.06 (0.96–1.17)
BCS + RT	1.21 (1.02–1.42)	0.80 (0.71–0.9)	0.62 (0.57–0.68)	0.63 (0.58–0.68)	0.62 (0.57–0.68)	0.61 (0.56–0.67)
Endocrine therapy^b^						
No	1 (Ref.)	1 (Ref.)	1 (Ref.)	1 (Ref.)	1 (Ref.)	1 (Ref.)
Yes	0.89 (0.74–1.06)	1.01 (0.89–1.14)	1.12 (1.02–1.23)	1.13 (1.04–1.24)	1.14 (1.04–1.25)	1.15 (1.05–1.26)

*ALND* axillary lymph node dissection, *BCS* breast-conserving surgery, *ER* estrogen receptor, *RT* radiotherapy, *SNB* sentinel node biopsy

^a^Hazard ratios were adjusted for chemotherapy, radiotherapy/surgery, endocrine therapy, tumor size, lymph node involvement, axillary lymph node dissection, ER status, age at diagnosis, level of education, prior sick leave, region of residency, and calendar year of diagnosis. The underlying time-scale was time since diagnosis

^b^Initiation of endocrine therapy

Table 3 shows the average number of days spent on sick leave for a typical patient (covariate pattern), selected to represent a large proportion of women (age 50–54 years at diagnosis, ER+ tumor, no lymph node involvement, tumor size 1–30 mm, SNB only, period of diagnosis 2009–2012, Stockholm-Gotland region, middle education, no prior sick leave). During the first 5 years after diagnosis, women who had received chemotherapy in addition to breast-conserving therapy spent on average 182 (95% CI 151–218) additional days on sick leave compared with women not receiving chemotherapy, but with otherwise similar characteristics. Correspondingly, women who initiated endocrine therapy spent 30 (95% CI 18–44) additional days on sick leave, and women treated with both endocrine therapy and chemotherapy 210 (95% CI 185–239) additional days compared with women not receiving any of these treatments. Among women treated with mastectomy, radiotherapy was associated with 53 (95% CI 37–69) additional days on sick leave. Overall, results were similar when selecting other covariate patterns (Supplementary Table 5).

**Table 3:** Mean length of stay on sick leave within the first 5 years of diagnosis by type of treatment

Treatment	Length of stay on sick leave	Difference in length of stay on sick leave
	Days^a^ (95% CI)	
Breast-conserving surgery		
+ RT	102 (87–116)	Ref.
+ RT + ET	132 (123–141)	30 (18–44)
+ CHEMO + RT	284 (249–324)	182 (151–218)
+ CHEMO + RT + ET	312 (288–338)	210 (185–239)
Mastectomy		
Mastectomy only	123 (101–144)	Ref.
+ ET	144 (129–157)	21 (3–38)
+ RT	176 (145–205)	53 (37–69)
+ CHEMO	303 (264–345)	180 (148–217)
+ CHEMO + ET	329 (305–352)	206 (180–233)
+ CHEMO + RT	368 (324–409)	245 (209–280)
+ CHEMO + RT + ET	397 (372–419)	274 (246–300)

*ET* initiation of endocrine therapy, *CHEMO* chemotherapy, *RT* radiotherapy

^a^Estimates have been predicted holding all of the other variables constant, using the following covariate pattern: age 50–54 years at diagnosis, ER+ tumor, no lymph node involvement, tumor size 1–30 mm, SNB only, period of diagnosis 2009–2012, Stockholm-Gotland region, middle education, no prior sick leave

**Fig. 1:**
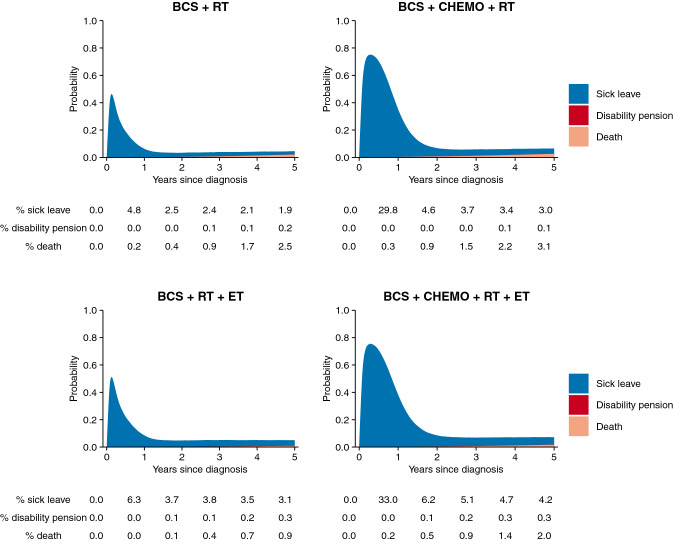
Predicted probabilities of sick leave, disability pension, and death by treatment modality in women treated with breast-conserving surgery followed by radiotherapy. (Estimates have been predicted holding all of the other variables constant, using the following covariate pattern: age 50–54 years at diagnosis, ER+ tumor, no lymph node involvement, tumor size 1–30 mm, SNB only, period of diagnosis 2009–2012, Stockholm-Gotland region, middle education, no prior sick leave.) *BCS* breast-conserving treatment, *ET* initiation of endocrine therapy, *CHEMO* chemotherapy, *RT* radiotherapy

Using the same typical patient as specified above, Fig. 1 shows the predicted probabilities of sick leave, disability pension receipt, and death by type of oncological treatment in women treated with breast-conserving therapy. (Corresponding probabilities for women treated with mastectomy are presented in Supplementary Fig. 3). In the first year after diagnosis, the probability of sick leave was highest in women receiving chemotherapy. From year 2 onward, absolute differences in predicted probabilities of sick leave between treatment types were small.

In adjusted analyses of cause-specific sick leave, both chemotherapy and endocrine therapy were associated with increased rates of sick leave due to depression or anxiety (Table 4). The risk associated with endocrine treatment was highest in the first years following diagnosis (HR year 2 1.72, 95% CI 1.06–2.77), whereas the risk associated with chemotherapy was highest at year 5 (HR 1.50, 95% CI 1.05–2.14). The pattern was similar when restricting the analysis to women with ER+ tumors only (Supplementary Table 6). In absolute terms, using the same typical patient as above, endocrine therapy was associated with on average 9 (95% CI 4–16) additional days on sick leave due to depression or anxiety during the first 5 years after diagnosis (Supplementary Table 7). Chemotherapy was associated with 5 (95% CI – 6 to 17) additional days.

**Table 4:** Hazard ratios and 95% confidence intervals for cause-specific sick leave

Treatment	Cause-specific sick leave					
	Month 6	Year 1	Year 2	Year 3	Year 4	Year 5
	HR^a^ (95% CI)	HR^a^ (95% CI)	HR^a^ (95% CI)	HR^a^ (95% CI)	HR^a^ (95% CI)	HR^a^ (95% CI)
Sick leave due to depression or anxiety						
Chemotherapy						
No	1 (Ref.)	1 (Ref.)	1 (Ref.)	1 (Ref.)	1 (Ref.)	1 (Ref.)
Yes	1.04 (0.77–1.42)	1.09 (0.83–1.42)	1.14 (0.83–1.58)	1.25 (0.97–1.61)	1.38 (1.05–1.80)	1.50 (1.05–2.14)
Radiotherapy (RT)						
Mastectomy only	1 (Ref.)	1 (Ref.)	1 (Ref.)	1 (Ref.)	1 (Ref.)	1 (Ref.)
Mastectomy + RT	0.85 (0.52–1.38)	0.96 (0.66–1.41)	1.10 (0.71–1.72)	1.06 (0.76–1.49)	0.99 (0.70–1.41)	0.94 (0.57–1.55)
BCS + RT	0.73 (0.50–1.07)	0.83 (0.61–1.13)	0.95 (0.66–1.39)	0.97 (0.74–1.29)	0.98 (0.73–1.31)	0.99 (0.66–1.49)
Endocrine therapy^b^						
No	1 (Ref.)	1 (Ref.)	1 (Ref.)	1 (Ref.)	1 (Ref.)	1 (Ref.)
Yes	1.65 (1.05–2.61)	1.69 (1.12–2.57)	1.72 (1.06–2.77)	1.62 (1.08–2.45)	1.52 (0.99–2.33)	1.44 (0.84–2.44)
Sick leave due to cancer						
Chemotherapy						
No	1 (Ref.)	1 (Ref.)	1 (Ref.)	1 (Ref.)	1 (Ref.)	1 (Ref.)
Yes	1.97 (1.70–2.28)	1.80 (1.63–2.00)	1.68 (1.53–1.85)	1.63 (1.48–1.80)	1.61 (1.45–1.78)	1.60 (1.44–1.77)
Radiotherapy (RT)						
Mastectomy only	1 (Ref.)	1 (Ref.)	1 (Ref.)	1 (Ref.)	1 (Ref.)	1 (Ref.)
Mastectomy + RT	1.69 (1.38–2.07)	1.51 (1.30–1.74)	1.37 (1.17–1.61)	1.31 (1.10–1.58)	1.29 (1.05–1.57)	1.27 (1.03–1.57)
BCS + RT	1.57 (1.31–1.87)	0.98 (0.87–1.11)	0.55 (0.47–0.64)	0.37 (0.29–0.47)	0.29 (0.21–0.40)	0.24 (0.16–0.36)
Endocrine therapy^b^						
No	1 (Ref.)	1 (Ref.)	1 (Ref.)	1 (Ref.)	1 (Ref.)	1 (Ref.)
Yes	0.87 (0.72–1.05)	0.94 (0.82–1.07)	0.98 (0.87–1.12)	1.00 (0.88–1.15)	1.01 (0.88–1.17)	1.02 (0.88–1.18)
Sick leave due to other reasons						
Chemotherapy						
No	1 (Ref.)	1 (Ref.)	1 (Ref.)	1 (Ref.)	1 (Ref.)	1 (Ref.)
Yes	1.03 (0.87–1.22)	1.07 (0.94–1.23)	1.11 (0.96–1.28)	1.09 (0.96–1.24)	1.04 (0.90–1.20)	1.01 (0.85–1.20)
Radiotherapy (RT)						
Mastectomy only	1 (Ref.)	1 (Ref.)	1 (Ref.)	1 (Ref.)	1 (Ref.)	1 (Ref.)
Mastectomy + RT	0.77 (0.60–0.99)	0.93 (0.78–1.10)	1.06 (0.89–1.27)	1.10 (0.93–1.29)	1.05 (0.88–1.25)	1.00 (0.80–1.25)
BCS + RT	0.53 (0.43–0.64)	0.56 (0.49–0.65)	0.61 (0.52–0.71)	0.67 (0.59–0.77)	0.74 (0.64–0.85)	0.77 (0.64–0.93)
Endocrine therapy^b^						
No	1 (Ref.)	1 (Ref.)	1 (Ref.)	1 (Ref.)	1 (Ref.)	1 (Ref.)
Yes	1.05 (0.84–1.32)	1.18 (0.98–1.42)	1.26 (1.03–1.53)	1.26 (1.04–1.52)	1.20 (0.97–1.46)	1.14 (0.90–1.46)

*BCS* breast-conserving therapy, *RT* radiotherapy

^a^Hazard ratios were adjusted for chemotherapy, radiotherapy/surgery, endocrine therapy, tumor size, lymph node involvement, axillary lymph node dissection, ER status, age at diagnosis, level of education, prior sick leave, region of residency, calendar year of diagnosis, and medical history of mental disorders. The underlying time-scale was time since diagnosis

^b^Initiation of endocrine therapy

## Discussion

In this population-based study of women with early-stage breast cancer, chemotherapy had the largest impact on working life. During the first 5 years after diagnosis, we found that a typical woman aged 50–54 years at diagnosis who received chemotherapy on average spent around 180 additional days on sick leave as a result of treatment, of which usually 126 days (6 × 21 days) can be attributed to active treatment and a few weeks to waiting time between surgery and start of treatment. Initiation of endocrine therapy was associated with around 30 additional sick-leave days, and post-mastectomy radiotherapy with around 50 days (of which typically 35 days [5 weeks] can be attributed to active treatment). Both chemotherapy and endocrine therapy were associated with long-term increased rates of sick leave, although absolute differences were small.

The risk of absence from work after treatment for breast cancer has been explored in several systematic reviews [8, 21, 22]. Most recently, a meta-analysis found that receipt of chemotherapy was significantly associated with unemployment (odds ratio [OR] 1.41, 95% CI, 1.14–1.73), whereas no such associations were observed for radiotherapy (OR 1.10, 95% CI 0.91–1.34) or endocrine therapy (OR 0.96, 95% CI 0.89–1.04) [22]. However, this meta-analysis involved pooling of studies with not only different work-related outcomes, but also different lengths of follow-up, ranging from 1 month to 10 years. While it is obvious from the current literature that chemotherapy adversely influences the ability to work in the first year of diagnosis irrespective of the measure used, these pooled estimates do not allow for differentiation between short- and long-term consequences on working life.

Besides three Swedish studies [2, 5, 7], we know of only a few studies presenting long-term data on the relationship between oncological treatment and sick leave and/or disability pension [6, 9, 23]. Corroborating results from two previous Swedish studies [2, 5] and a Dutch study [6] but contrasting a Danish study [9], we found that chemotherapy was associated with an increased risk of sick leave up to 5 years after diagnosis. In contrast to what is suggested from the above-mentioned meta-analysis, we also found evidence of an increased risk of sick leave in women initiating endocrine therapy. Our findings are in agreement with three previous Swedish studies [2, 5, 24], of which one was a randomized controlled trial [24]. Endocrine therapy was not associated with increased risk of disability pension in the Dutch study [6], but as pointed out by the authors, women receiving endocrine therapy have a better prognosis and may because of that be in less need of disability benefits. In the present study, post-mastectomy radiotherapy was not associated with a long-term increased risk of sick leave.

Previous research has demonstrated that women with breast cancer are at increased risk of both depression [25] and absence from work due to depression [26]. The interplay between possible contributing factors, including the role of cancer treatment, is not fully understood. In our current analysis, we found that receipt of chemotherapy and initiation of endocrine therapy were associated with increased rates of sick leave due depression or anxiety. Whereas an impact of chemotherapy on psychological well-being has been reported previously in premenopausal women [27, 28], large placebo-controlled trials have not found evidence of associations between tamoxifen or aromatase inhibitors and depression or mental health [29, 30]. Hence, the cause of this finding is unclear and needs further investigation.

Similar to most other studies in this area, confounding by indication is a potential issue. Despite restricting the analysis to women with early-stage tumors and controlling for a broad range of variables related to prognosis, both receipt of chemotherapy and radiotherapy were positively associated with death, suggesting that confounding by indication is not fully accounted for. It is probable that the observed hazard ratios, in particular several years out from diagnosis, in part can be explained by breast cancer recurrences. A further limitation, also shared with many earlier studies, was that our analysis relied on data from a small group of women not initiating endocrine therapy, increasing the uncertainty of findings. In addition, no data were available on sick-leave periods 14 days or shorter, possibly resulting in an underestimated proportion of women on sick leave. Further, our cause-specific analyses are limited by lack of information on secondary sick-leave diagnoses. For example, the main cause “breast cancer” has likely been used for many different cancer-related conditions.

Despite these limitations, our study is one of the largest studies on breast cancer and absence from work to date, using high-quality data from several national population-based registers with virtually complete follow-up. To our knowledge, our study is the first to provide adjusted estimates on the absolute impact of oncological treatment for breast cancer on absence from work. We have previously presented estimates of the permanent loss of working time after a breast cancer diagnosis [7], but in our current approach, we have also been able to take temporary absence such as sick leave into account. These estimates are particularly compelling since they are not only easy to understand, but also provide a complete picture of the burden of breast cancer treatment on subsequent working life.

In conclusion, our findings demonstrate that although the majority of women treated for early-stage breast cancer can return to and remain in work, oncological treatments are associated with increased risks of prolonged and recurrent sick leave. Women treated with chemotherapy are at a particular high risk, with a considerable amount of time lost from work due to treatment. Bearing in mind that chemotherapy is a lifesaving treatment, this is often an acceptable consequence. However, in light of the ongoing debate about overtreatment of early-stage breast cancer, our findings further emphasize the importance of identifying women likely to benefit from chemotherapy to avoid unnecessary sick leave and associated individual and societal cost. Furthermore, the long-term increased risk of sick leave warrants attention from caregivers taking part in breast cancer rehabilitation.

## Electronic supplementary material

Below is the link to the electronic supplementary material.

Supplementary file1 (PDF 321 kb)

## Data Availability

The datasets generated and/or analyzed during the current study are not publicly available due to confidentiality reasons regulated by Swedish law. Data can be requested from the register holder.
