# Supplementary material for: Impact of chemotherapy, radiotherapy, and endocrine therapy on sick leave in women with early-stage breast cancer during a 5-year period: a population-based cohort study
Source: Breast Cancer Res Treat. 2020 Jun 6;182(3):699–707. doi: 10.1007/s10549-020-05720-4 (PMC7320921; doi:10.1007/s10549-020-05720-4)
Supplement: Supplementary file 1 — Supplementary file1 (PDF 321 kb) [file 10549_2020_5720_MOESM1_ESM.pdf]

Impact of chemotherapy, radiotherapy, and endocrine therapy on sick leave in women with early-stage breast cancer during a five-year period - a population-based cohort study

Supplementary material

Supplemental Figure 1

Illustration of the multi-state model with arrows representing possible transitions. n refers to the number of events for each transition

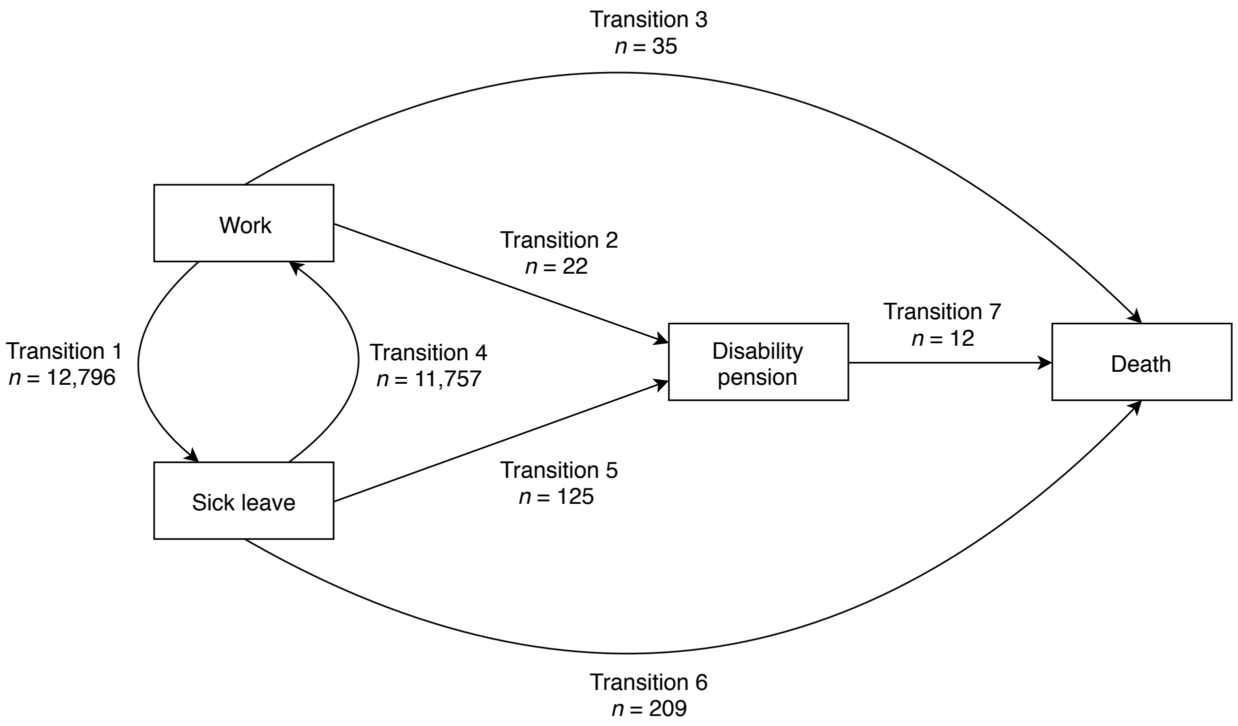

Supplementary Table 1

Model specification for prediction of absolute measures for the all-cause model (Supplemental Figure 1)

Transition	df for baseline hazard function	df for time-dependent effects	Variables with time-dependent effects
Transition 1 (work to sick leave)	5	3	Chemotherapy, mastectomy + radiotherapy, breast-conserving surgery + radiotherapy, endocrine therapy
Transition 2 (work to disability pension)	1	0	
Transition 3 (work to death)	1	0	
Transition 4 (sick leave to work)	5	1-3	Chemotherapy (3 df), mastectomy + radiotherapy (3 df), breast-conserving surgery + radiotherapy (1 df), endocrine therapy (3 df)
Transition 5 (sick leave to disability pension)	3	0	
Transition 6 (sick leave to death)	3	0	
Transition 7 (disability pension to death)	1	0	

Selection of knots was guided by the AIC/BIC.

The following variables were adjusted for: chemotherapy (yes, no), surgery/radiotherapy (mastectomy, mastectomy + radiotherapy, breast-conserving surgery + radiotherapy), initiation of endocrine therapy (yes, no), tumor size (1-30, >30 mm), lymph node involvement (N0, N1), type of axillary surgery (SNB only, ALND), ER status (negative, positive), age at diagnosis (categorized in five-year intervals), highest level of education [low ($\leq$ 9 years), middle (10-12 years), high ($\geq$ 13 years)], prior sick leave defined as a record of sick leave (>14 days) in the period 366 to 730 days before diagnosis (yes, no), calendar year of diagnosis (2005-2008, 2009-2012), and region of residence (Stockholm-Gotland, Uppsala-Orebro, the Northern regions). Interaction terms were included between type of surgery and type of oncological treatment, as well as between chemotherapy and endocrine therapy.

Supplemental Figure 2

Illustration of the cause-specific multi-state model with arrows representing possible transitions. n refers to the number of events for each transition

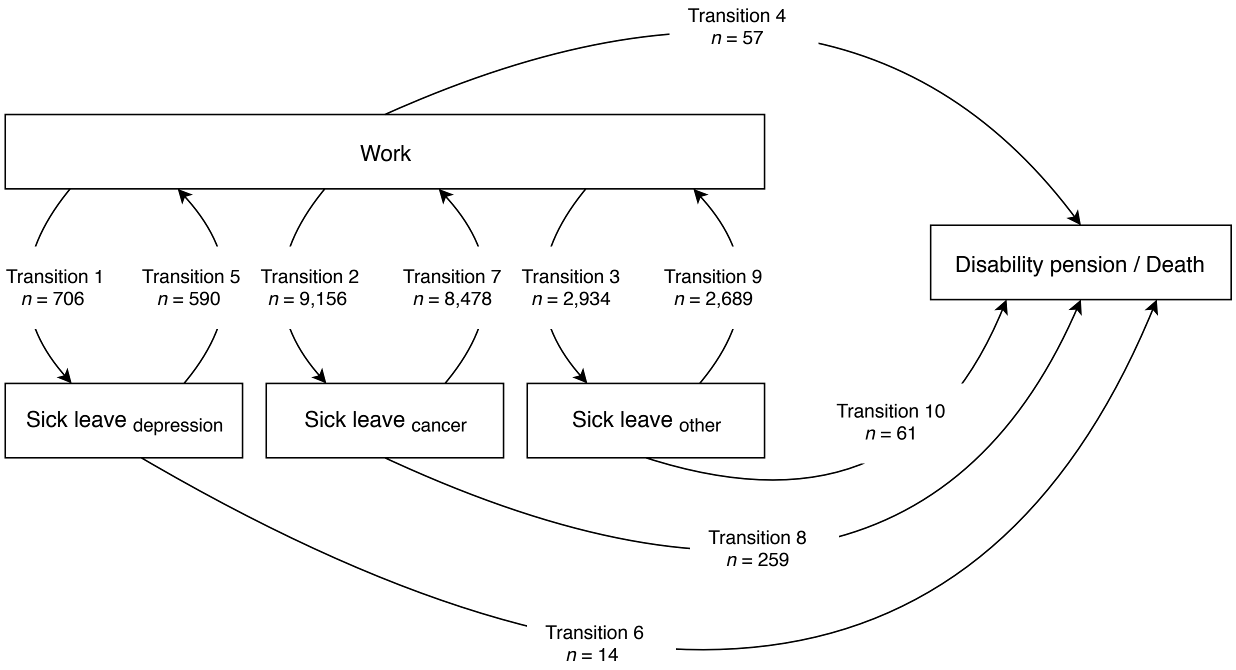

Supplementary Table 2

Model specification for prediction of absolute measures for the cause-specific model (Supplemental Figure 2)

Transition	df for baseline hazard function	df for time-dependent effects	Variables with time-dependent effects
Transition 1 (work to sick leave depression)	3	0	
Transition 2 (work to sick leave cancer)	3	1	Chemotherapy, mastectomy + radiotherapy, breast-conserving surgery + radiotherapy, endocrine therapy
Transition 3 (work to sick leave other)	3	0	
Transition 4 (work to dp/death)	1	0	
Transition 5 (sick leave depression to work)	3	0	
Transition 6 (sick leave depression to dp/death)	1	0	
Transition 7 (sick leave cancer to work)	3	1	Chemotherapy, mastectomy + radiotherapy, breast-conserving surgery + radiotherapy, endocrine therapy
Transition 8 (sick leave cancer to dp/death)	3	0	
Transition 9 (sick leave other to work)	3	1	Chemotherapy, mastectomy + radiotherapy, breast-conserving surgery + radiotherapy, endocrine therapy
Transition 10 (sick leave other to dp/death)	1	0	

Selection of knots was guided by the AIC/BIC.

The following variables were adjusted for: chemotherapy (yes, no), surgery/radiotherapy (mastectomy, mastectomy + radiotherapy, breast-conserving surgery + radiotherapy), initiation of endocrine therapy (yes, no), tumor size (1-30, >30 mm), lymph node involvement (N0, N1), type of axillary surgery (SNB only, ALND), ER status (negative, positive), age at diagnosis (categorized in five-year intervals), highest level of education [low ($\leq$ 9 years), middle (10-12 years), high ($\geq$ 13 years)], prior sick leave defined as a record of sick leave (>14 days) in the period 366 to 730 days before diagnosis (yes, no), calendar year of diagnosis (2005-2008, 2009-2012), region of residence (Stockholm-Gotland, Uppsala-Orebro, the Northern regions) and prior mental disorders (yes, no). (The model for transition 6 did only include tumor characteristics and the model for transition 10 did only include tumor and treatment characteristics due to few events). An interaction term was included between chemotherapy and endocrine therapy in the models for transitions 1-3, 5, and 7-9.

Supplementary Table 3

Hazard ratios and 95% confidence intervals for sick leave and return to work after sick leave in women with **ER+ tumors** only

Variable	Label	Sick_leave	Return_to_work
Chemotherapy	No	1 (Ref.)	1 (Ref.)
	Yes	1.27 (1.20-1.33)	0.42 (0.40-0.44)
Radiotherapy	Mastectomy	1 (Ref.)	1 (Ref.)
	Mastectomy + RT	1.13 (1.05-1.21)	0.83 (0.78-0.90)
	Breast-cons. + RT	0.82 (0.78-0.87)	0.91 (0.86-0.96)
Endocrine therapy	No	1 (Ref.)	1 (Ref.)
	Yes	1.10 (1.01-1.19)	0.92 (0.85-1.00)
Tumor size (mm)	<10	1 (Ref.)	1 (Ref.)
	10-20	1.04 (0.98-1.09)	0.86 (0.82-0.91)
	>20	1.08 (1.01-1.15)	0.83 (0.78-0.88)
Lymph nodes	N0	1 (Ref.)	1 (Ref.)
	N1	1.02 (0.96-1.08)	0.92 (0.87-0.98)
Axillary surgery	SNB only	1 (Ref.)	1 (Ref.)
	ALND	1.08 (1.02-1.13)	0.85 (0.80-0.90)
Age	<45	1 (Ref.)	1 (Ref.)
	45-49	0.90 (0.84-0.96)	1.11 (1.04-1.19)
	50-54	0.95 (0.89-1.01)	1.15 (1.07-1.22)
	55-59	0.92 (0.86-0.98)	1.16 (1.08-1.24)
	60-64	0.60 (0.56-0.64)	1.28 (1.19-1.38)
Education	Low (0-9 yr)	1 (Ref.)	1 (Ref.)
	Middle (10-12 yr)	1.20 (1.11-1.28)	1.10 (1.02-1.19)
	High (> 12 yr)	1.11 (1.04-1.20)	1.09 (1.01-1.18)
Prior sick leave	No	1 (Ref.)	1 (Ref.)
	Yes	1.56 (1.47-1.65)	0.76 (0.71-0.81)
Region	Stockholm-Got.	1 (Ref.)	1 (Ref.)
	Uppsala-Orebro	1.13 (1.08-1.18)	1.24 (1.18-1.30)
	Norra	1.23 (1.16-1.31)	0.94 (0.88-1.00)
Year of diagnosis	2005-2008	1 (Ref.)	1 (Ref.)
	2009-2012	0.93 (0.89-0.97)	1.19 (1.14-1.25)

Hazard ratios were adjusted for chemotherapy, radiotherapy/surgery, endocrine therapy, tumor size, lymph node involvement, axillary lymph node dissection, age at diagnosis, level of education, prior sick leave, region of residency, and calendar year of diagnosis.

Supplementary Table 4

Time-dependent hazard ratios and 95% confidence intervals for sick leave in women with **ER+ tumors** only

Treatment	Label	6 months	Year 1	Year 2	Year 3	Year 4	Year 5
Chemotherapy	No	1 (Ref.)	1 (Ref.)	1 (Ref.)	1 (Ref.)	1 (Ref.)	1 (Ref.)
	Yes	1.78 (1.54-2.06)	1.44 (1.29-1.59)	1.17 (1.08-1.27)	1.15 (1.06-1.24)	1.13 (1.05-1.22)	1.12 (1.03-1.21)
Radiotherapy	Mastectomy	1 (Ref.)	1 (Ref.)	1 (Ref.)	1 (Ref.)	1 (Ref.)	1 (Ref.)
	Mastectomy + RT	1.4 (1.11-1.77)	1.16 (0.98-1.38)	1.02 (0.91-1.15)	1.02 (0.91-1.13)	1.01 (0.9-1.12)	1 (0.89-1.12)
	BCS + RT	1.31 (1.09-1.57)	0.76 (0.66-0.87)	0.55 (0.49-0.62)	0.57 (0.51-0.63)	0.56 (0.5-0.62)	0.55 (0.5-0.62)
Endocrine therapy	No	1 (Ref.)	1 (Ref.)	1 (Ref.)	1 (Ref.)	1 (Ref.)	1 (Ref.)
	Yes	0.84 (0.66-1.07)	1.12 (0.95-1.32)	1.26 (1.13-1.41)	1.27 (1.14-1.41)	1.28 (1.15-1.43)	1.3 (1.16-1.46)

Hazard ratios were adjusted for chemotherapy, radiotherapy/surgery, endocrine therapy, tumor size, lymph node involvement, axillary lymph node dissection, age at diagnosis, level of education, prior sick leave, region of residency, and calendar year of diagnosis.

Supplementary Table 5

Length of stay on sick leave during the first five years after diagnosis by different covariate patterns

Age <45 years at diagnosis, ER+ tumor, no lymph node involvement, tumor size 1-30 mm, SNB only, period of diagnosis 2009-2012, Stockholm-Gotland region, middle education, no prior sick leave:

Treatment	Length of stay on sick leave, days (95% CI)	Difference (95% CI)
BCS + RT	121 (104-138)	Ref.
BCS + RT + ET	158 (147-169)	37 (21-52)
BCS + CHEMO + RT	319 (278-362)	198 (163-235)
BCS + CHEMO + RT + ET	351 (324-381)	230 (202-260)
MAST	146 (120-172)	Ref.
MAST + ET	170 (154-186)	24 (3-46)
MAST + RT	205 (169-239)	59 (41-76)
MAST + CHEMO	341 (298-386)	195 (159-233)
MAST + CHEMO + ET	369 (343-396)	223 (195-253)
MAST + CHEMO + RT	408 (361-453)	262 (224-299)
MAST + CHEMO + RT + ET	441 (414-467)	295 (265-324)

Age 45-49 years at diagnosis, ER+ tumor, no lymph node involvement, tumor size 1-30 mm, SNB only, period of diagnosis 2009-2012, Stockholm-Gotland region, middle education, no prior sick leave:

Treatment	Length of stay on sick leave, days (95% CI)	Difference (95% CI)
BCS + RT	105 (90-119)	Ref.
BCS + RT + ET	136 (126-145)	31 (18-45)
BCS + CHEMO + RT	288 (253-330)	183 (153-220)
BCS + CHEMO + RT + ET	318 (291-346)	213 (186-243)
MAST	126 (105-148)	Ref.
MAST + ET	147 (133-161)	21 (3-39)
MAST + RT	180 (150-210)	54 (37-70)
MAST + CHEMO	308 (269-351)	182 (150-219)
MAST + CHEMO + ET	334 (309-359)	208 (181-236)
MAST + CHEMO + RT	375 (331-416)	249 (212-283)
MAST + CHEMO + RT + ET	403 (378-426)	277 (248-304)

Age 50-54 years at diagnosis, ER+ tumor, no lymph node involvement, tumor size 1-30 mm, SNB only, period of diagnosis 2009-2012, Stockholm-Gotland region, middle education, no prior sick leave:

Treatment	Length of stay on sick leave, days (95% CI)	Difference (95% CI)
BCS + RT	102 (87-116)	Ref.
BCS + RT + ET	132 (123-141)	30 (18-44)
BCS + CHEMO + RT	284 (249-324)	182 (151-218)
BCS + CHEMO + RT + ET	312 (288-338)	210 (185-239)
MAST	123 (101-144)	Ref.
MAST + ET	144 (129-157)	21 (3-38)
MAST + RT	176 (145-205)	53 (37-69)
MAST + CHEMO	303 (264-345)	180 (148-217)

Treatment	Length of stay on sick leave, days (95% CI)	Difference (95% CI)
MAST + CHEMO + ET	329 (305-352)	206 (180-233)
MAST + CHEMO + RT	368 (324-409)	245 (209-280)
MAST + CHEMO + RT + ET	397 (372-419)	274 (246-300)

Age 55-59 years at diagnosis, ER+ tumor, no lymph node involvement, tumor size 1-30 mm, SNB only, period of diagnosis 2009-2012, Stockholm-Gotland region, middle education, no prior sick leave:

Treatment	Length of stay on sick leave, days (95% CI)	Difference (95% CI)
BCS + RT	98 (84-111)	Ref.
BCS + RT + ET	127 (118-135)	30 (17-42)
BCS + CHEMO + RT	277 (242-317)	179 (149-214)
BCS + CHEMO + RT + ET	304 (279-330)	206 (180-233)
MAST	119 (97-139)	Ref.
MAST + ET	138 (124-151)	19 (3-37)
MAST + RT	170 (140-199)	51 (35-67)
MAST + CHEMO	296 (257-337)	177 (146-212)
MAST + CHEMO + ET	319 (297-343)	200 (175-228)
MAST + CHEMO + RT	359 (317-399)	241 (206-274)
MAST + CHEMO + RT + ET	387 (363-408)	268 (241-293)

Age 60-64 years at diagnosis, ER+ tumor, no lymph node involvement, tumor size 1-30 mm, SNB only, period of diagnosis 2009-2012, Stockholm-Gotland region, middle education, no prior sick leave:

Treatment	Length of stay on sick leave, days (95% CI)	Difference (95% CI)
BCS + RT	63 (54-72)	Ref.
BCS + RT + ET	82 (75-89)	19 (11-28)
BCS + CHEMO + RT	205 (177-236)	141 (116-170)
BCS + CHEMO + RT + ET	223 (203-244)	160 (140-182)
MAST	77 (62-91)	Ref.
MAST + ET	90 (80-100)	13 (2-25)
MAST + RT	115 (93-137)	38 (26-51)
MAST + CHEMO	216 (187-250)	140 (115-169)
MAST + CHEMO + ET	234 (215-254)	157 (138-178)
MAST + CHEMO + RT	272 (237-305)	196 (165-223)
MAST + CHEMO + RT + ET	291 (270-311)	215 (194-235)

Age 50-54 years at diagnosis, ER+ tumor, no lymph node involvement, tumor size 1-30 mm, SNB only, period of diagnosis 2009-2012, **Stockholm-Gotland region**, middle education, no prior sick leave:

Treatment	Length of stay on sick leave, days (95% CI)	Difference (95% CI)
BCS + RT	102 (87-116)	Ref.
BCS + RT + ET	132 (123-141)	30 (18-44)
BCS + CHEMO + RT	284 (249-324)	182 (151-218)
BCS + CHEMO + RT + ET	312 (288-338)	210 (185-239)
MAST	123 (101-144)	Ref.
MAST + ET	144 (129-157)	21 (3-38)
MAST + RT	176 (145-205)	53 (37-69)
MAST + CHEMO	303 (264-345)	180 (148-217)
MAST + CHEMO + ET	329 (305-352)	206 (180-233)
MAST + CHEMO + RT	368 (324-409)	245 (209-280)
MAST + CHEMO + RT + ET	397 (372-419)	274 (246-300)

Age 50-54 years at diagnosis, ER+ tumor, no lymph node involvement, tumor size 1-30 mm, SNB only, period of diagnosis 2009-2012, **Uppsala-Orebro region**, middle education, no prior sick leave:

Treatment	Length of stay on sick leave, days (95% CI)	Difference (95% CI)
BCS + RT	94 (82-106)	Ref.
BCS + RT + ET	122 (113-130)	27 (15-39)
BCS + CHEMO + RT	270 (237-308)	176 (146-210)
BCS + CHEMO + RT + ET	296 (272-322)	202 (176-228)
MAST	114 (95-133)	Ref.
MAST + ET	132 (121-145)	18 (2-35)
MAST + RT	165 (139-190)	51 (36-65)
MAST + CHEMO	289 (252-329)	175 (142-210)
MAST + CHEMO + ET	310 (289-334)	196 (171-224)
MAST + CHEMO + RT	352 (313-387)	237 (204-269)
MAST + CHEMO + RT + ET	377 (356-397)	263 (237-288)

Age 50-54 years at diagnosis, ER+ tumor, no lymph node involvement, tumor size 1-30 mm, SNB only, period of diagnosis 2009-2012, **Northern region**, middle education, no prior sick leave:

Treatment	Length of stay on sick leave, days (95% CI)	Difference (95% CI)
BCS + RT	136 (116-155)	Ref.
BCS + RT + ET	175 (160-192)	39 (22-58)
BCS + CHEMO + RT	341 (302-387)	205 (172-246)
BCS + CHEMO + RT + ET	378 (346-410)	241 (210-276)
MAST	163 (134-192)	Ref.
MAST + ET	190 (170-209)	26 (3-50)
MAST + RT	225 (187-263)	62 (43-81)
MAST + CHEMO	367 (320-416)	204 (166-244)
MAST + CHEMO + ET	396 (368-428)	233 (201-268)
MAST + CHEMO + RT	436 (386-484)	273 (233-312)
MAST + CHEMO + RT + ET	471 (440-501)	308 (274-340)

Supplemental Figure 3

Predicted probabilities of sick leave, disability pension, and death by treatment modality for women treated with **mastectomy**. (Estimates have been predicted holding all of the other variables constant, using the following covariate pattern: age 50-54 years at diagnosis, ER+ tumor, no lymph node involvement, tumor size 1-30 mm, SNB only, period of diagnosis 2009-2012, Stockholm-Gotland region, middle education, no prior sick leave.) Abbreviations: ET, endocrine therapy; CHEMO, chemotherapy; RT, radiotherapy

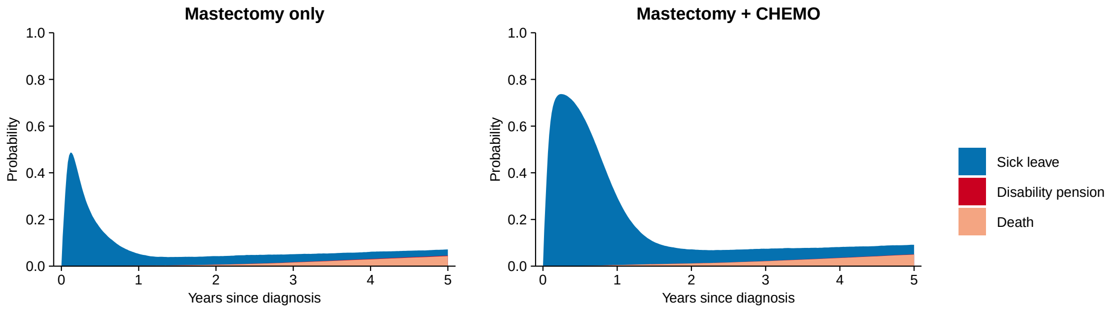

% sick leave	0.0	5.1	3.6	3.4	3.1	2.6	0.0	29.0	6.0	5.2	4.7	4.1
% disability pension	0.0	0.0	0.0	0.1	0.2	0.2	0.0	0.0	0.0	0.1	0.1	0.1
% death	0.0	0.2	0.6	1.6	2.9	4.3	0.0	0.5	1.2	2.1	3.5	4.9

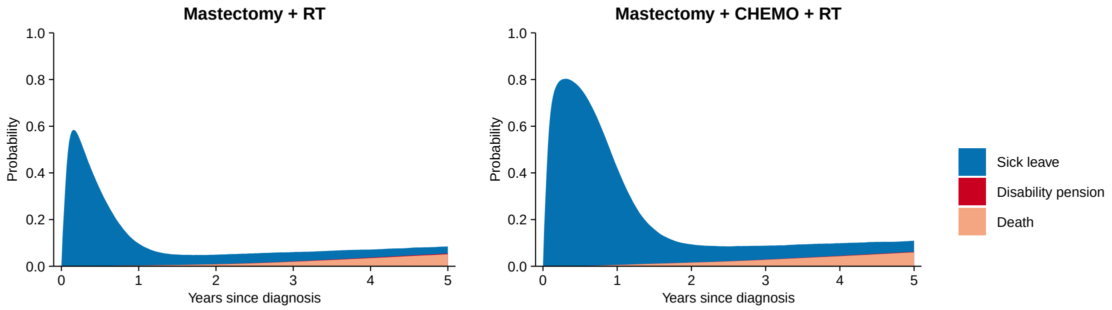

% sick leave	0.0	9.5	4.1	3.9	3.4	3.0	0.0	41.7	7.8	5.9	5.4	4.8
% disability pension	0.0	0.0	0.1	0.1	0.2	0.3	0.0	0.0	0.0	0.1	0.2	0.2
% death	0.0	0.3	0.9	2.0	3.5	5.2	0.0	0.6	1.6	2.8	4.3	6.0

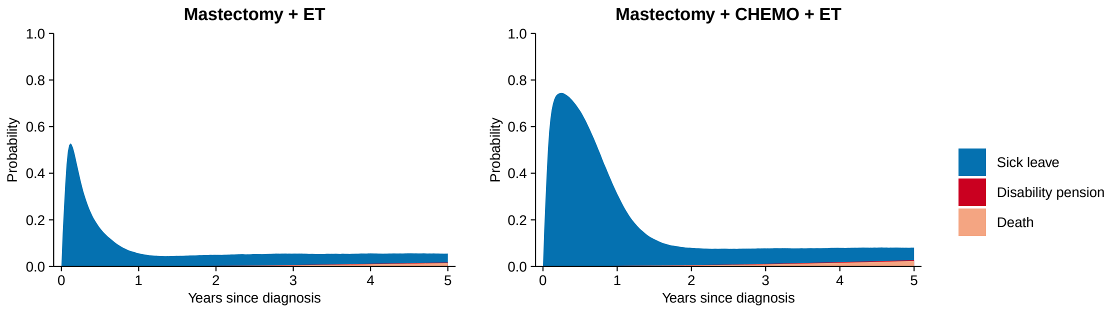

% sick leave	0.0	5.4	4.7	4.9	4.3	3.8	0.0	31.0	7.4	6.6	6.1	5.4
% disability pension	0.0	0.0	0.0	0.1	0.2	0.2	0.0	0.0	0.1	0.2	0.3	0.3
% death	0.0	0.1	0.2	0.5	0.9	1.4	0.0	0.2	0.5	0.9	1.6	2.4

Supplementary Table 6

Hazard ratios and 95% confidence intervals for cause-specific sick leave in women with **ER+ tumors** only

Sick leave due to **depression or anxiety**

Treatment	Label	6 months	Year 1	Year 2	Year 3	Year 4	Year 5
Chemotherapy	No	1 (Ref.)	1 (Ref.)	1 (Ref.)	1 (Ref.)	1 (Ref.)	1 (Ref.)
	Yes	0.87 (0.62-1.22)	0.95 (0.7-1.28)	1.06 (0.73-1.53)	1.18 (0.9-1.53)	1.33 (1.01-1.74)	1.48 (1.07-2.06)
Radiotherapy	Mastectomy	1 (Ref.)	1 (Ref.)	1 (Ref.)	1 (Ref.)	1 (Ref.)	1 (Ref.)
	Mastectomy + RT	0.91 (0.55-1.5)	0.91 (0.59-1.41)	0.95 (0.55-1.65)	1.03 (0.73-1.46)	1.1 (0.79-1.54)	1.16 (0.77-1.76)
	BCS + RT	0.7 (0.47-1.05)	0.76 (0.54-1.07)	0.84 (0.54-1.32)	0.89 (0.67-1.19)	0.94 (0.71-1.25)	0.99 (0.69-1.43)
Endocrine therapy	No	1 (Ref.)	1 (Ref.)	1 (Ref.)	1 (Ref.)	1 (Ref.)	1 (Ref.)
	Yes	1.8 (1.01-3.23)	2.06 (1.15-3.71)	2.19 (1.09-4.4)	1.58 (0.99-2.53)	1.1 (0.65-1.86)	0.8 (0.39-1.68)

Sick leave due to **cancer**

Treatment	Label	6 months	Year 1	Year 2	Year 3	Year 4	Year 5
Chemotherapy	No	1 (Ref.)	1 (Ref.)	1 (Ref.)	1 (Ref.)	1 (Ref.)	1 (Ref.)
	Yes	1.97 (1.69-2.28)	1.78 (1.6-1.98)	1.63 (1.48-1.79)	1.57 (1.42-1.73)	1.54 (1.39-1.7)	1.52 (1.37-1.69)
Radiotherapy	Mastectomy	1 (Ref.)	1 (Ref.)	1 (Ref.)	1 (Ref.)	1 (Ref.)	1 (Ref.)
	Mastectomy + RT	1.84 (1.48-2.28)	1.54 (1.31-1.8)	1.3 (1.08-1.56)	1.2 (0.97-1.49)	1.14 (0.9-1.45)	1.11 (0.86-1.44)
	BCS + RT	1.59 (1.32-1.92)	0.95 (0.83-1.09)	0.46 (0.38-0.56)	0.27 (0.2-0.36)	0.17 (0.11-0.27)	0.11 (0.06-0.23)
Endocrine therapy	No	1 (Ref.)	1 (Ref.)	1 (Ref.)	1 (Ref.)	1 (Ref.)	1 (Ref.)
	Yes	0.88 (0.69-1.12)	1.01 (0.85-1.19)	1.1 (0.97-1.26)	1.15 (1.01-1.31)	1.17 (1.02-1.34)	1.18 (1.03-1.36)

Sick leave due to **other reasons**

Treatment	Label	6 months	Year 1	Year 2	Year 3	Year 4	Year 5
Chemotherapy	No	1 (Ref.)	1 (Ref.)	1 (Ref.)	1 (Ref.)	1 (Ref.)	1 (Ref.)
	Yes	0.94 (0.79-1.13)	1 (0.86-1.15)	1.03 (0.89-1.2)	1.06 (0.93-1.21)	1.07 (0.94-1.23)	1.08 (0.91-1.27)
Radiotherapy	Mastectomy	1 (Ref.)	1 (Ref.)	1 (Ref.)	1 (Ref.)	1 (Ref.)	1 (Ref.)
	Mastectomy + RT	0.81 (0.62-1.05)	0.91 (0.75-1.1)	0.99 (0.81-1.2)	1.03 (0.86-1.22)	1.03 (0.86-1.22)	1.02 (0.82-1.26)
	BCS + RT	0.55 (0.45-0.67)	0.57 (0.49-0.66)	0.59 (0.5-0.7)	0.65 (0.57-0.75)	0.73 (0.63-0.84)	0.77 (0.65-0.92)
Endocrine therapy	No	1 (Ref.)	1 (Ref.)	1 (Ref.)	1 (Ref.)	1 (Ref.)	1 (Ref.)
	Yes	1.23 (0.93-1.62)	1.36 (1.08-1.71)	1.41 (1.11-1.81)	1.26 (1.02-1.56)	1.02 (0.8-1.31)	0.9 (0.65-1.23)

Hazard ratios were adjusted for chemotherapy, radiotherapy/surgery, endocrine therapy, tumor size, lymph node involvement, axillary lymph node dissection, age at diagnosis, level of education, prior sick leave, region of residency, calendar year of diagnosis, and medical history of mental disorders.

Supplementary Table 7

Length of stay on sick leave due to **depression or anxiety** during the first five years after diagnosis

(Predictions were made for the following covariate pattern: age 50-54 years at diagnosis, ER+ tumor, no lymph node involvement, tumor size 1-30 mm, SNB only, period of diagnosis 2009-2012, Stockholm-Gotland region, middle education, no prior sick leave)

Treatment	Length of stay on sick leave, days (95% CI)	Difference (95% CI)
BCS + RT	8 (4-13)	Ref.
BCS + RT + ET	18 (13-24)	9 (4-16)
BCS + CHEMO + RT	13 (3-26)	5 (-6-17)
BCS + CHEMO + RT + ET	22 (14-30)	13 (6-21)
MAST	9 (3-15)	Ref.
MAST + ET	18 (11-27)	10 (3-17)
MAST + RT	11 (4-19)	2 (-2-7)
MAST + CHEMO	14 (1-29)	5 (-7-19)
MAST + CHEMO + ET	22 (13-32)	13 (6-22)
MAST + CHEMO + RT	17 (2-35)	9 (-6-26)
MAST + CHEMO + RT + ET	27 (16-41)	19 (7-32)
